# Interactions Between Neurotrophins and Ovarian Steroids in Endometriosis and Their Implications for Neuroangiogenesis: A Narrative Review

**DOI:** 10.3390/cimb48070649

**Published:** 2026-06-24

**Authors:** Olivia Tania Hernández-Hernández, Dora María Velázquez-Hernández, Ignacio Camacho-Arroyo

**Affiliations:** 1 Secretaría de Ciencia, Humanidades, Tecnología e Innovación-Unidad de Investigación en Reproducción Humana, Instituto Nacional de Perinatología-Facultad de Química, Universidad Nacional Autónoma de México, Ciudad de México 11000, Mexico; taniaohh2@gmail.com; 2 Unidad de Investigación en Reproducción Humana, Instituto Nacional de Perinatología-Facultad de Química, Universidad Nacional Autónoma de México, Ciudad de México 11000, Mexico

**Keywords:** endometriosis, progesterone, estradiol, nerve growth factor, brain-derived neurotrophic factor, neuroangiogenesis, pain

## Abstract

Endometriosis is a long-term gynecological condition marked by the growth of endometrial-like tissue outside the uterus, which undergoes proliferation, bleeding, and regeneration. This disease is associated with disrupted steroid hormone signaling, notably progesterone (P4) resistance and estradiol (E2) dominance. P4 resistance has been associated with impaired activation of the progesterone receptor (PR) and reduced transcription of P4 target genes, while elevated E2 levels induce estrogen receptor (ER)-mediated signaling, enhancing estrogen-dependent lesion growth. This hormonal imbalance contributes to a pro-inflammatory microenvironment, chronic pelvic pain, infertility, and enhanced neuroangiogenesis. Emerging evidence indicates that the coordinated regulation of neurotrophins and sex hormones promotes nerve fibers and blood vessel growth and invasion within endometriotic lesions. P4 and E2 have been shown to modulate the expression of key neurotrophins, including nerve growth factor (NGF) and brain-derived neurotrophic factor (BDNF). This review presents current evidence on the interplay between neurotrophins and ovarian steroids in endometriosis, with a specific focus on their contribution to neuroangiogenesis and pain pathophysiology. The review includes articles in English containing the Medical Subject Headings (MeSH) terms: “endometriosis”, “neurotrophins”, “nerve growth factor”, “brain-derived neurotrophic factor”, “neuroangiogenesis”, “progesterone”, and “estradiol”, found in the PubMed database published between 2000 and 24 May 2026. This review included a range of original research articles, systematic reviews, meta-analyses, prospective observational studies, case–control studies, and review papers, for a total of 122 articles.

## 1. Introduction

Endometriosis is an estrogen-dependent gynecological disorder characterized by the presence of endometrial-like tissue (lesions) that proliferates, bleeds, and regenerates outside the uterine cavity and the eutopic endometrium. Endometriotic lesions are predominantly found in the peritoneum, ovary, myometrium (adenomyosis), and bowel [1]. This disease represents one of the most important causes of infertility, chronic pelvic pain, dysmenorrhea, dyspareunia, and dysuria, affecting 10–15% of women of reproductive age, significantly impacting their quality of life [2]. Diagnosis typically takes 5 to 12 years from the onset [3]. The gold standard for diagnosing endometriosis is surgical visualization, typically via laparoscopy, followed by histological confirmation of excised lesions [4]. Current therapies include three classes of treatments: (1) analgesics to manage symptoms, (2) hormonal therapies designed to inhibit estrogen-dependent growth of lesions, and (3) laparoscopic surgical excision [5].

Several hypotheses have been proposed to explain the origin of endometriotic lesions. The most accepted theory is retrograde menstruation, based on trans-tubal reflux and the implantation of endometrial tissue originally eutopic in ectopic sites [4]. The tissue injury and repair hypothesis suggests that repeated microtrauma at the endometrial–myometrial interface may initiate estrogen-dependent repair mechanisms that contribute to lesion development [6]. Once established, these lesions elicit a chronic inflammatory response that leads to fibrosis, scar formation, and adhesions [7]. The disorder is characterized by functional endometrial-like tissue implants that proliferate and invade organs outside the uterine cavity [5]. Clinically, endometriosis is classified into three main subtypes according to lesion location: superficial peritoneal endometriosis, ovarian endometriomas, and deep infiltrating endometriosis [8].

Endometriotic lesions cause persistent local inflammation, leading to the production of prostaglandins, cytokines, and growth factors [9]. Interestingly, ectopic endometriotic implants recruit their own neural and vascular supplies via neuroangiogenesis, a process of new blood vessel formation that occurs simultaneously with the growth of new neurons [10]. The factors responsible for neurogenesis are incompletely identified. However, neurotrophins, such as nerve growth factor (NGF) and brain-derived neurotrophic factor (BDNF), are overexpressed in endometriomas compared with the eutopic endometrium [11].

In endometriosis, the growth of endometrial tissue outside the uterine cavity disrupts signaling by progesterone (P4) and estradiol (E2), resulting in P4 resistance and E2 dominance. This hormone imbalance leads to heightened inflammation, pelvic pain, neuroangiogenesis, and infertility [12].

We performed a literature search in PubMed (https://pubmed.ncbi.nlm.nih.gov) to identify English-language articles on neurotrophins, sex hormones, neuroangiogenesis, and endometriosis. We applied a combination of Medical Subject Headings (MeSH) terms to screen and select relevant studies: “endometriosis”, “neurotrophins”, “nerve growth factor”, “brain-derived neurotrophic Factor”, “neuroangiogenesis”, “progesterone”, and “estradiol”. After the search, studies published from 2000 to 24 May 2026 were classified according to the health-specific parameters of the text. Titles and abstracts were reviewed to select articles for full-text evaluation. This review included a range of original research articles, systematic reviews, meta-analyses, prospective observational studies, case–control studies, and review articles, for a total of 122 articles.

## 2. Dysregulation of Progesterone and Estrogen Signaling in Endometriosis

P4 and E2 act in a compartment-specific manner within the stroma and epithelium to maintain endometrial homeostasis and support female fertility. Disruption of the balance between epithelial and stromal P4 and E2 signaling can lead to progesterone resistance and estrogen dominance, processes strongly associated with the development and progression of endometriosis [12].

### 2.1. Progesterone Resistance in Endometriosis

P4 resistance refers to a tissue that fails to respond appropriately to hormone exposure. In endometriosis, this resistance is characterized by impaired activation of the intracellular progesterone receptor (PR) and reduced transcription of progesterone-responsive target genes [13,14].

Loss of P4 responsiveness has significant consequences in both endometriotic lesions and the eutopic endometrium of women with endometriosis. P4 signaling is essential for counteracting E2-induced cell proliferation and inflammation and for promoting decidualization. This implies that P4 resistance may increase lesion growth and lead to a non-receptive endometrium.

In addition, local E2 levels are elevated in endometriosis through several mechanisms, including upregulation of the E2-synthesizing enzyme aromatase p450, reduced expression of 17-hydroxysteroid dehydrogenase type 2, which is usually induced by P4 to convert E2 into the less potent estrone, but is diminished under P4-resistant conditions, and altered expression of estrogen receptors (ERs) [15].

P4 exerts its effects by interacting with PR or membrane P4 receptors (mPRs), thereby regulating the transcription of its target genes. PR is expressed as two functionally distinct isoforms, PR-A and PR-B, transcribed from two promoters within the same gene. In humans, PR-A lacks 164 amino acids relative to PR-B [16]. It has been shown that PR-A is the isoform required for uterine functions and is sufficient for fertility, while PR-B promotes uterine epithelial proliferation when PR-A is absent [17]. Interestingly, PR-A overexpression leads to endometrial hyperproliferation and infertility, revealing the importance of the PR-A/PR-B ratio for an adequate response to P4 [18]. mPRs and the P4 receptor membrane components (PGRMCs) mediate P4 non-classical (also known as non-genomic) actions [19]. mPRs and PGRMCs are expressed in female reproductive tissues, such as the endometrium, myometrium, ovaries, and placenta [20,21].

In endometriotic lesions, P4 resistance is closely associated with the loss or modification of PR expression [22,23] and in the eutopic endometrium of women with endometriosis [23]. A study found that in women with endometriosis, PR levels remained consistent throughout the menstrual cycle [24]. Other studies have reported low PR-B (typically associated with stimulatory P4 actions) expression, with the predominant isoform in endometriotic lesions being PR-A (mainly inhibitory) [23]. Interestingly, PR-A can suppress the transcriptional activity mediated by PR-B [14]. Notably, PR-B deficiency is more pronounced than in the eutopic endometrium of women with endometriosis.

The activation of mPRs is essential for achieving the full effects of P4 in responsive cells, since P4 effects are only partially dependent on PR activation. The content and functionality of these receptors are altered in diseases such as cancer [25,26]. The expression pattern of mPRs is tissue-specific, and their activation by P4 regulates signaling pathways involved in ovulation and the maintenance of pregnancy [27]. mPRs are expressed in female reproductive and embryonic tissues [19,28]. Data from our laboratory indicate that, in the eutopic and ectopic endometrium of women with endometriosis, the expression of mPRγ, mPRδ, mPRα, and mPRβ is downregulated. Furthermore, in the ectopic endometrium of patients with endometriosis, the levels of mPRα and mPRβ proteins are reduced, supporting the hypothesis that P4 resistance contributes to the etiology of endometriosis [20]. In another study conducted by our research group, we demonstrated that mPRβ is expressed at lower levels in endometrial tissues than in tissues from women without endometriosis. Moreover, P4, by activating mPRβ, controls decidualization of endometrial stromal cells in women with and without endometriosis. Furthermore, small interfering Ribonucleic Acid (RNA) transfection targeting mPRβ caused a 60–80% reduction in the expression of decidualization markers in control cells and a 60–70% reduction in eutopic cells from women with endometriosis, confirming that mPRβ is essential for the decidualization process in cells from both healthy women and women with endometriosis [29]. Defects in decidualization and embryo implantation have been associated with recurrent pregnancy loss, preeclampsia, and infertility [30].

Progestins offer therapeutic effects that extend beyond endocrine suppression. In endometriotic lesions, they influence key molecular pathways that sustain them, including inflammation, angiogenesis, extracellular matrix remodeling, and nerve sensitivity. These effects are primarily mediated by PR signaling, which is disrupted in endometriosis—especially due to reduced PR-B expression and an altered PR-A/PR-B ratio. The extent to which progestins can activate the remaining PR-B or compensate via PR-A-dependent pathways may partly influence their efficacy [14].

Among currently available agents, dienogest (DNG) is the most prescribed because it is safe and effective for the treatment of endometriosis [31]. DNG, a 19-nortestosterone derivative, has shown sustained efficacy in reducing endometriosis-associated pain [31]. DNG activates both PR-A and PR-B isoforms, leading to atrophy of ectopic endometrial cells. It exhibits minimal androgenic or corticosteroid-like effects and inhibits the transcription of various mediators involved in lesion survival and pain. These include aromatase, cyclooxygenase-2, interleukin-6 (IL-6), interleukin-8 (IL-8), NGF, and vascular endothelial growth factor (VEGF) in human endometriotic epithelial cells [32]. The downregulation of NGF and VEGF by DNG could reduce neuroangiogenesis, a central process in lesion innervation and chronic pelvic pain [32].

Selective progesterone receptor modulators (SPRMs) are synthetic molecules that interact with the PR. By binding to PR, SPRMs act as agonists or antagonists, although mixed activity has also been observed [33]. The effect of the interaction between SPRMs and PR depends on several factors, including the amounts of PR-A and PR-B in each tissue and receptor conformation changes induced by different ligands [33]. The interest in developing an SPRM for use in endometriosis lies in its selective inhibition of endometrial proliferation without causing estrogen deprivation or its undesirable side effects, in pain relief through the suppression of prostaglandin and cytokine synthesis in endometrial cells, and in its action on PRs expressed in the spiral arterioles, which helps reduce endometrial bleeding [34].

A systematic review of 10 randomized controlled trials involving 960 women evaluated the efficacy and safety of SPRMs for relieving pain associated with endometriosis. Mifepristone was more effective than placebo for dysmenorrhea and dyspareunia, at least at doses of 5 or 10 mg. Some women reported amenorrhea and hot flashes as side effects; however, the authors noted that there is insufficient evidence to draw firm conclusions regarding the safety and efficacy of SPRMs for the treatment of endometriosis [35].

### 2.2. E2 Dominance in Endometriosis

The endometrium is the primary target tissue for E2, which acts via ERs to induce mucosal proliferation during the proliferative phase and to promote PR synthesis, thereby preparing the endometrium for the secretory phase [36].

E2 acts on female reproduction through two intracellular receptors (ERα and ERβ) and the membrane-bound G protein-coupled estrogen receptor 1 (GPER). There are two classical or genomic mechanisms of action. First, E2 passively enters the cell membrane by diffusion and interacts with ERα and ERβ located in the cytoplasm, inducing the formation of dimers that translocate to the nucleus and directly bind estrogen response elements (EREs) or transcription factors, thereby recruiting co-regulatory proteins and chromatin remodeling factors to activate or inhibit transcription of target genes. Secondly, ER can regulate genes that lack canonical EREs. ER can modify gene expression by interacting with transcription factors, such as activator protein-1 (AP-1), specificity protein-1 (SP-1), and nuclear factor kappa-B (NF-κB), which can bind DNA [36]. The hormone-independent mechanism of action occurs when ER regulates E2 responses by activating growth factor signaling via phosphorylation. In non-classical regulation, E2 binds GPER in the membrane, leading to various non-genomic responses. GPER activates metalloproteinases and induces the release of heparin-binding epidermal growth factor, which binds and activates epidermal growth factor receptor. This interaction leads to the activation of downstream signaling molecules such as extracellular signal-regulated kinase 1/2 (ERK1/2), followed by the production of cyclic adenosine monophosphate (cAMP), intracellular calcium mobilization, and phosphoinositide-3-kinase (PI3K) activation [37].

Studies using knockout mouse models have demonstrated the critical role of ERα and ERβ in reproduction [36]. ERα-deficient female mice are infertile due to uterine unresponsiveness to E2, whereas ERβ-deficient females are subfertile and exhibit impaired ovulation, highlighting distinct but complementary roles of each receptor subtype [36]. In the normal endometrium, E2, derived from the circulation, exerts its primary effect on ERα, while ERβ and GPER exhibit reduced expression, thereby supporting the central role of E2 in mediating uterine proliferative responses [36]. However, ER messenger RNA (mRNA) and protein expression differ between the normal healthy endometrium and ectopic and eutopic endometrial lesions. In endometrial lesions, ERβ expression is increased, whereas ERα expression is decreased [38]. The increase in ERβ expression may be due to pathological hypomethylation of the ERβ promoter in endometriotic stromal cells, thereby making it hyperactive [38]. Additionally, it has been determined that ERβ suppresses ERα expression, thereby increasing ERβ dominance in the ERβ/ERα ratio in endometriotic cells [39]. This shift in receptor dominance has significant functional consequences, as ERβ has been implicated in promoting inflammatory signaling, cell survival, and resistance to P4 action [40].

It has also been suggested that reduced ERα expression leads to decreased PR expression in endometriotic stromal cells. PR is known to be an ERα target gene in several cell types, including malignant breast epithelial cells. While PR promoter sequences respond to E2, neither contains a classic palindromic ERE sequence. However, several non-classical regulatory elements (e.g., activator protein 1 and specificity protein 1) have been identified in the human PR promoter, and ERα binding has been observed at these sites [41,42]. These events can lead to PR loss and P4 resistance in endometriosis.

GPER mediates rapid estrogen effects [43]. In humans, several studies have reported GPER mRNA and protein expression in uterine epithelial and stromal cells, the endometrium, the myometrium, and the early pregnancy decidua [44]. An increase in GPR30 (the previous term of GPER) mRNA levels was reported in the endometrium of women without endometriosis during the proliferative phase of the menstrual cycle. In contrast, higher expression of GPR30 was observed in the ectopic endometrium of women with endometriosis during the secretory phase. It has been suggested that elevated GPR30 expression plays a relevant role in the progression of endometriosis [45]. Given the positive association between GPER and proliferation, and the role of estrogens in promoting endometriotic growth and survival, it is speculated that the abnormally elevated GPER expression in the secretory endometrial epithelium of women with endometriosis contributes to the proliferation of detached endometriotic cells in the peritoneal cavity [46].

**Figure 1:**
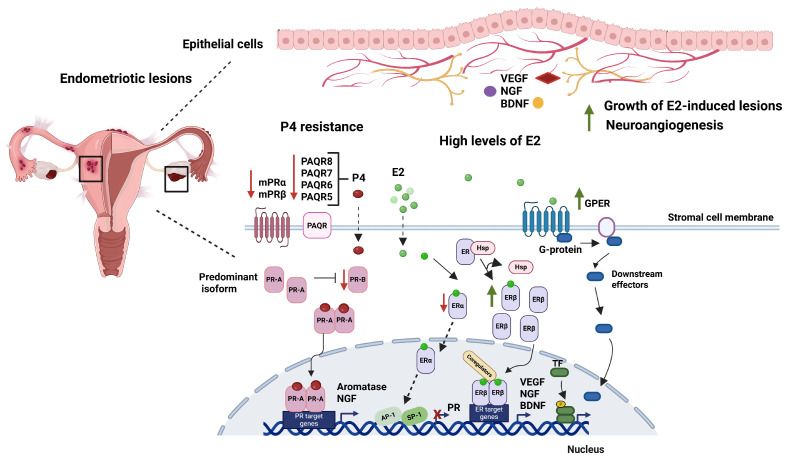
Dysregulation of progesterone (P4) and estradiol (E2) signaling in endometriosis. When the balance between epithelial–stromal P4 and E2 signaling is disrupted, P4 resistance and E2 dominance are likely to develop, leading to endometriosis. P4 resistance is characterized by failure to activate its receptors. P4 exerts its effects by interacting with intracellular (PR) or membrane receptors. PRs are expressed as two functionally distinct isoforms, PR-A and PR-B. Membrane receptors are divided into two groups: membrane progesterone receptors (mPRs), which belong to the progesterone and adipoQ receptor (PAQR) family, and progesterone receptor membrane components (PGRMCs). Expression of PR-B, mPRα, mPRβ, PAQR7, PAQR8, PAQR5, and PAQR6 is reduced in endometriotic lesions. P4 signaling is required to counteract E2-induced cell proliferation. E2 exerts its effects through two intracellular receptors (ERα and ERβ) and the membrane receptor G protein-coupled estrogen receptor 1 (GPER). ERβ and GPER are required for the development of endometriotic lesions. Reduced ERα expression leads to decreased PR expression. E2 may provide trophic support to nerve fibers by regulating neurotrophins production, including nerve growth factor (NGF) and brain-derived neurotrophic factor (BDNF), in the endometrium of women with endometriosis. P4 treatment increased NGF and aromatase expression. Created in BioRender, Zamora, C. (2026). https://BioRender.com/y2nrqk5 (accessed on 14 May 2026).

A positive link has been suggested between E2 signaling and pain mechanisms in endometriosis. E2 appears to modulate sensory and sympathetic innervation in peritoneal endometriosis and in the uterus of rodents and humans [47]. It has been reported that E2 regulates the synthesis and release of NGF [48], VEGF [49], and BDNF [50]. Furthermore, hormone therapies designed to counter the E2 excess in endometriosis have been shown to decrease the density of endometrial nerve fibers in women with endometriosis, suggesting a role for E2 in increased innervation (Figure 1) [51].

Therapies that reduce E2 action are used to treat endometriosis and other proliferative endometrial disorders. Selective estrogen receptor modulators (SERMs) are synthetic molecules that bind to ER. The function of these molecules depends on the cellular context, acting as either agonists or antagonists. Tamoxifen, raloxifene, and bazedoxifene have anti-proliferative effects and regress endometriotic implants [52]. In experimental models, SERMs directly affected endometrial blood vessels and suppressed prostaglandin production in the endometrium without exhibiting the systemic effects of estrogen deprivation. Raloxifene, a pharmaceutical compound used to treat postmenopausal osteoporosis, exerted an anti-estrogenic effect on rat uterine tissue, leading to regression of endometriotic implants [53]. In a murine model, bazedoxifene was an effective treatment for endometriosis, as evidenced by reduced endometriotic implant size and decreased proliferating cell nuclear antigen levels, a marker of endometrial proliferation. Regression of endometriotic lesions appears to have occurred primarily through the suppression of estrogen-induced proliferation [54]. This mechanism involves both suppression of ER expression and direct antagonism of ER by this SERM.

## 3. Neurotrophins

Neurotrophins are a family of soluble polypeptide growth factors that regulate neuronal development and the survival of different neuronal populations. Their expression is not restricted to neuronal tissue; they are also found in platelets and reproductive tissues, including the ovaries, endometrium, and myometrium [55]. The key components of the neurotrophins system in humans are NGF, BDNF, neurotrophins-3 (NT-3), -4/5 (NT-4/5), as well as their respective receptors, the high-affinity transmembrane tropomyosin receptor kinase (TrkA, TrkB, TrkC), members of the Trk proto-oncogene family and the low-affinity p75 neurotrophin receptor (p75NTR), a member of the tumor necrosis factor (TNF) receptor superfamily [56]. Neurotrophins are synthesized as preproproteins in the endoplasmic reticulum; the signal peptide is cleaved off, and the pro-neurotrophins are secreted. Before their release from the cell, pro-precursors are proteolytically processed by convertase subtilisin/kexin enzymes, which dimerize to yield their mature forms in the trans-Golgi network [55]. Pro- and mature neurotrophins initiate their biological actions by binding to Trk receptors. Trk receptors contain an intracellular tyrosine kinase domain and an extracellular domain that contains a leucine- and cysteine-rich motif responsible for ligand binding [57].

Neurotrophins are known to promote the growth, differentiation, and function of nerve cells. However, NGF and BDNF have also been shown to regulate other functions in various tissues, including the proliferation and migration of vascular cells and the secretion of pro-inflammatory cytokines [5]. The role of neurotrophins in endometrial function is particularly important for axon growth and nerve fiber sprouting [58]. The overexpression of neurotrophins has been linked to reproductive pathologies, including premature ovarian failure [59], endometrial cancer [60], and endometriosis [47]. In endometriosis, nerve fibers have been found near the endometriotic epithelium and stroma, a process accompanied by angiogenesis [61].

NGF exerts significant influence on biological functions, including the extension and maintenance of sympathetic and sensory nerve fibers, inflammatory and neuropathic pain, oxidative stress, and successful pregnancy [62]. It has also been found that NGF protein levels are higher in women with endometriosis than in women without endometriosis [63].

BDNF plays a key role in both nociceptive and neuropathic pain. It is involved in the development and persistence of chronic pain in various chronic disorders [64]. BDNF and its TrkB receptor are expressed at significant levels in the reproductive system of female mammals [65]. As demonstrated by Browne (2012), BDNF mRNA and protein levels were significantly higher in women with endometriosis compared with women without endometriosis [63]. High levels of BDNF in peritoneal fluid have also been observed and positively correlate with pelvic pain in women with endometriosis compared to the control group [66].

### 3.1. Neurotrophin Receptors

Neurotrophins initiate signal transduction through ligand-induced receptor dimerization, internalization, and activation of their specific receptors via phosphorylation of the kinase domain in the cytoplasmic region. Mature neurotrophins also bind to Trk, resulting in prosurvival or trophic signaling, and to p75NTR, which promotes cell death [59]. The Trk family comprises three members: TrkA, TrkB, and TrkC. Each one has preferred neurotrophin-binding partner(s): TrkA binds preferentially NGF, TrkB binds BDNF and NT-4/5, and TrkC binds NT-3. In addition, all neurotrophins activate p75NTR. The extracellular domain of Trk consists of a cysteine-rich cluster, followed by three leucine-rich repeats, another cysteine-rich cluster, and two Ig-like domains. All receptors have a single transmembrane region that terminates in a cytoplasmic tyrosine kinase domain, which is flanked by several tyrosine residues that serve as phosphorylation-dependent docking sites for cytoplasmic adaptors and enzymes [57].

It is known that intracellular signaling activated by neurotrophins–Trk is involved in the physiological and pathological neuronal aspects affecting cell viability, synaptic function, and neurogenesis [67]. The most studied signaling pathways activated by Trk receptors include the ERK/MAPK pathway, the PI3K/Akt pathway, and the phosphoinositide phospholipase C-γ/protein kinase C-γ pathway. These pathways regulate processes such as cell proliferation, neuronal survival, differentiation, and neurite outgrowth. Additionally, p75NTR is known to stimulate cell death via apoptotic signaling pathways [68].

NGF and BDNF participate in pain syndromes, including osteoarthritis, rheumatoid arthritis, and fibromyalgia [69,70]. Neurotrophins levels are also linked to endometriosis-associated pain. Alterations in NGF/TrkA signaling contribute to chronic pain and inflammation [71]. Recent studies have found that BDNF, in combination with TrkB, contributes to the development of endometriosis and clinically relevant symptoms. Abnormalities in ectopic endometrial apoptosis and defects in autophagic function are associated with the pathogenesis of endometriosis. A study showed that TrkB inhibits apoptosis in the ectopic endometrium. Upon binding to BDNF, TrkB can activate the PI3K signaling pathway, enhance AKT activity, inhibit the activation of pro-apoptotic proteins, activate the anti-apoptotic protein Bcl-2, and prevent ectopic endometrial apoptosis [72]. The release of BDNF in the spinal dorsal horn is subsequently followed by binding to the TrkB receptor, culminating in central sensitization, which is associated with chronic pelvic pain and dysmenorrhea linked to endometriosis [72].

The formation of new blood vessels is a critical step in the progression of endometriosis. BDNF promotes angiogenesis via BDNF/TrkB signaling, which activates AKT phosphorylation in vascular endothelial cells, thereby enhancing angiogenesis [73]. As evidence, the TrkB inhibitor K252a has been reported to block AKT phosphorylation, thereby reducing angiogenesis [73]. BDNF/TrkB signaling intersects with the MAPK/ERK pathway in ectopic endometrial stromal cells. This pathway regulates the expression of Cyclooxygenase-2, which in turn induces VEGF production, a potent stimulator of endothelial cell proliferation and angiogenesis [74].

### 3.2. Regulation of Neuroangiogenesis by NGF and BDNF

The endometrium has intrinsic angiogenic potential, and endometriotic lesions tend to grow in areas with abundant blood vessels. This suggests that angiogenesis is necessary for the development of endometriosis. Increased vasculature is indeed observed around implants, often in a radial or circumferential pattern. This feature can indicate the presence of endometriosis, even when the implant itself is not visible, as in puckered peritoneal lesions. Angiogenesis is essential for both implant establishment and the support of ongoing lesion growth and progression [10].

The survival and proliferation of migrated endometrial cells are dependent on a suitable peritoneal microenvironment. Angiogenesis and neurogenesis are believed to promote the development of endometriosis and pelvic pain associated with endometrial debris. Neuroangiogenesis, the growth of nerve supply in parallel blood vessels in lesions, is considered essential for the survival of transplanted tissues and metastatic tumors [75].

It has been proposed that coordinated stimulation of neurotrophic and angiotrophic mitogens promotes nerve and blood vessel invasion in endometriosis. The co-expression of these factors is termed neuroangiogenesis and may also predispose to ectopic implantation [63]. In endometriosis, VEGF expression increases, promoting the formation of new blood vessels within and around endometriotic lesions, thereby contributing to their survival [76]. It has also been suggested that NGF promotes angiogenesis by increasing VEGF mRNA and protein levels in rat ovarian-derived cells [77]. NGF has been shown to promote the growth of new blood vessels in the ovaries by increasing VEGF production via a TrkA- and extracellular signal-regulated kinase 2 (ERK2)-dependent pathway in vitro [78,79]. A study demonstrated that drospirenone, a progestin used to treat endometriosis, significantly reduces inflammatory cytokines (interleukins 6 and 8), NGF, and VEGF expression in human endometriotic stromal cells [80].

Some studies have reported that NGF and BDNF expressions are modulated by E2 and P4 [81,82]. In fertile women, plasma BDNF levels increased from the early follicular phase through day 14 of the menstrual cycle, reaching a preovulatory peak similar to that of E2. A second increase in BDNF levels occurred during the mid-luteal phase, coinciding with the rise in P4 levels, peaking on day 24. It is interesting to note that amenorrhoeic and postmenopausal women had lower plasma BDNF levels than fertile women. BDNF levels were positively correlated with E2 and P4 levels and negatively correlated with menopausal stage. Furthermore, hormone replacement therapy restored BDNF levels to those seen in fertile women during the follicular phase [83].

In a study of ovariectomized female C57BL/6 mice administered E2, P4, or E2 plus P4 for four days, E2 treatment increased levels of mature BDNF (proBDNF) and NGFR in the uterus [84]. In a murine model of endometriosis, E2-treated females showed blood vessels (CD31 marker) in close association with nerve fibers (PGP9.5 marker) in endometriotic lesions [85]. Furthermore, telomerase-immortalized human endometrial endothelial cells treated with the ERβ agonist 2,3-bis(4-hydroxyphenyl) propionitrile showed increased NGF expression via ERβ [86]. It has been suggested that, during wound repair, dermal microvascular endothelial cells secrete NGF, which is necessary for nerve regeneration [86]. Additionally, estrogens act through ERs to regulate the expression of several axonal guidance and neurotrophic factors that mediate the complex interactions between vessels and nerves in endometriosis [80].

In addition, it has been demonstrated that vessels can produce signals, including NGF, that guide nerves and support neurogenesis [87]. Innervation of endometriotic implants has been documented in all types of lesions in women and in experimental animal models of the disease [75]. It has been reported that the rat model of endometriosis meets the criteria for face and construct validity, thereby serving as a valuable tool for elucidating the development of the nerve and blood supply in endometriotic lesions. This endometriosis model is surgically induced through autotransplantation of uterine tissue biopsies into the abdominal cavity. Uterine transplants become vascularized, form cysts that exhibit rapid growth, and contain efferent sensory and sympathetic fibers, remaining viable for periods exceeding ten months. Rats presenting cysts demonstrate diminished fertility and heightened pelvic pain, conditions that are analogous to those observed in women with endometriosis. Furthermore, ectopic cysts in rats closely resemble human ectopic endometriotic growths; notably, both of them respond similarly to ovarian hormones. Additionally, these cysts synthesize and release substances characteristic of ectopic endometrial growths, such as pro-inflammatory cytokines and prostaglandins found in the peritoneal fluid of women with endometriosis [88,89]. Therefore, this rat model provides a suitable platform for investigating the fundamental mechanisms underlying the signs and symptoms of endometriosis.

### 3.3. The Role of NGF and BDNF in Neurogenesis and Their Association with Pain in Women with Endometriosis

Endometriosis in women can result in the following types of pain: cyclic pain, dysmenorrhea, nociceptive pain (e.g., dyspareunia), and chronic pelvic pain [9]. Research has demonstrated that chronic pelvic pain does not directly correlate with the size of the lesion or the severity of the disease [90]. In some instances, pain persists even after surgical removal of endometrial lesions, and chronic pain recurs in patients after 12 months [91].

It is known that nerve fibers are present in endometriotic lesions, deep invasive endometriosis [92], ovarian endometriomas [93], and peritoneal lesions [75]. It has been shown that the functional layer of the endometrium in women with endometriosis contains unmyelinated C-type nerve fibers [75]. The presence of these fibers may play a role in the mechanisms involved in the generation of pelvic pain signals in women with endometriosis [94]. The types of nerve fibers have been identified using neuronal markers such as substance P and calcitonin gene-related peptide [7]. In peritoneal lesions, their innervation and density are similar to those of the eutopic endometrium [75]. Nerve fibers are rarely present in the functional layer of the endometrium in women without endometriosis [95]. Endometrial mucosal tissue has been observed to have a low density of sensory innervation [85].

The data above suggest a connection between the presence of nerve fibers and the perception of pelvic pain in women with endometriosis. Notably, neurotrophins and their receptors are synthesized in situ within peritoneal ectopic lesions, suggesting a role in facilitating and maintaining nerve fiber growth [96]. Furthermore, because of their involvement in synaptic transmission, neurotrophins are postulated to regulate the central pain pathway, contributing to central sensitization in hyperalgesia and peripheral painful conditions such as endometriosis. Sensitivity to pelvic pain is partly mediated through afferent nerves originating in the endometrium, ovaries and myometrium [97].

As demonstrated by Wessels (2015), BDNF plays a pivotal regulatory role in the development and maintenance of pain across a range of chronic diseases [84]. Primary afferent fibers initiate the release of this neurotrophin, which mediates the transition from acute to chronic pain and its subsequent maintenance. This process is intricately linked to noxious signals. The release of BDNF in the spinal dorsal horn can be stimulated by noxious signals, leading to BDNF binding the TrkB receptor. This ultimately induces central sensitization, which is associated with chronic pelvic pain and dysmenorrhea associated with endometriosis, as evidenced by case–control studies [98].

A meta-analysis reported that BDNF expression levels were significantly higher in ectopic endometrial tissue from women with endometriosis than in that from women without the disease. Similarly, a meta-analysis of data from six studies indicated that serum BDNF levels were significantly higher in patients with endometriosis than in controls. Furthermore, an analysis of three studies showed significantly higher NGF levels in the endometrial tissue of women with endometriosis than in women without it. A study evaluating serum NGF levels also found significantly higher NGF levels in patients with endometriosis than in controls [97].

**Figure 2:**
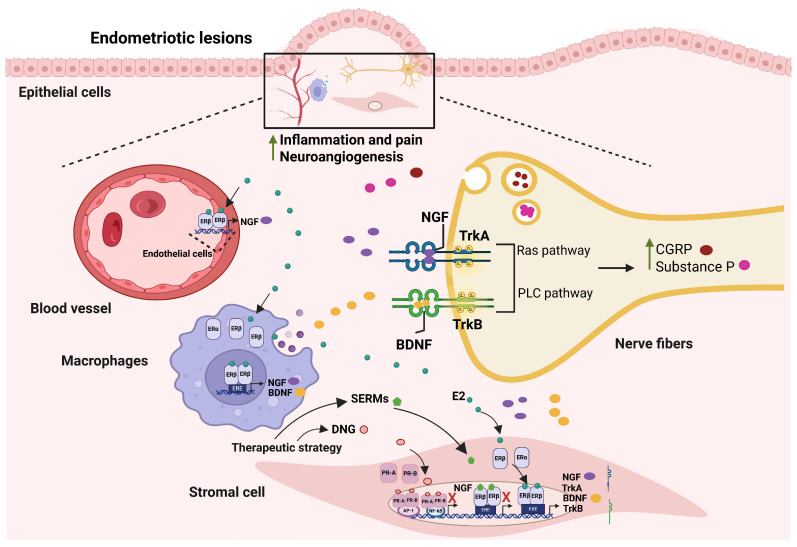
Interplay of neurotrophins and sex hormones in neuroangiogenesis regulation and their association with pain in women with endometriosis. In the disease, nerve fibers are found near epithelial and stromal cells and blood vessels. Neuroangiogenesis, the growth of nerve fibers in parallel blood vessels in lesions, is considered essential for the development of endometriosis and pelvic pain. Coordinated stimulation of neurotrophins and sex hormones promotes the invasion of nerves and blood vessels in endometriosis. Neurotrophins and their receptors are synthesized in situ within endometriotic lesions, facilitating and maintaining nerve fiber growth. Blood vessels can produce neurotrophins that guide nerves and support neurogenesis. The nerve growth factor (NGF)-tropomyosin receptor kinase A (TrkA) and brain-derived neurotrophic factor (BDNF)-tropomyosin receptor kinase B (TrkB) pathways increase the expression of neuropeptides that modulate central pain transmission, including substance P and calcitonin gene-related peptide (CGRP). NGF and BDNF can increase the number of sensory neurons involved in pain sensation. The presence of classical estrogen response elements (EREs) in the promoters of NGF, BDNF, TrkA, and TrkB suggests that estradiol (E2) modulates neurotrophins expression. E2, through Erβ, upregulates NGF expression in macrophages, which promote neurogenesis in endometriosis by coexisting with nerve fibers. Dienogest (DNG) downregulates various neuroangiogenic factors associated with endometriosis pain. Nuclear factor kappa-B (NF-κB) and activator protein-1 (AP-1) may contribute to the mechanism by which DNG inhibits NGF expression. Selective estrogen receptor modulators (SERMs) can be used to counteract excess E2 in endometriosis by decreasing nerve fiber density and blood vessel formation in women with the disease. Created in BioRender, Zamora, C. (2026). https://BioRender.com/up8ylny (accessed on 14 May 2026).

BDNF expression levels are significantly elevated in endometriosis patients suffering from chronic pelvic pain than in women without endometriosis. BDNF has been shown to increase the density and number of calcitonin gene-related peptide (CGRP)-containing nerve fibers, which are involved in inflammation and pain, thereby potentiating nociceptors and inducing pain. Furthermore, high CGRP expressions have been observed in the vicinity of endometriotic ectopic lesions [99] (Figure 2).

As demonstrated by Mechsner (2009) [94] and McKinnon (2012) [100], women with endometriosis who experienced elevated pain scores, specifically dysmenorrhea and pelvic pain, exhibited significantly higher concentrations of GAP-43 (a marker of neural outgrowth and regeneration) and protein gene-related product 9.5, a pan-neuronal marker of myelinated and unmyelinated nerve fibers, such as Aα, Aβ, Aγ, Aδ, B, and C fibers. Furthermore, a positive correlation was observed between severe dysmenorrhea and the presence of endometriosis-associated nerve fibers. Elevated NGF levels in patients with endometriosis were associated with a greater density of nerve supply. This, in turn, was associated with more severe pain [101].

It has been demonstrated that NGF increases the expression of neuropeptides that modulate central pain transmission, including substance P and CGRP [102]. It has been shown that NGF increases the number of sensory neurons involved in pain sensation. Furthermore, it has been associated with the occurrence of hyperalgesia and persistent inflammatory pain [103]. As demonstrated by Anaf (2006), NGF and TrkA expressions were increased in patients diagnosed with endometriosis [104]. Furthermore, the immunointensity of NGF and TrkA was higher in the endometriotic stroma and epithelium of women with endometriosis-associated deep dyspareunia than in those without deep dyspareunia [105].

Additionally, it was demonstrated that the expression of BDNF and TrKB in ectopic ovarian lesions and eutopic endometrium increases according to the stage of disease progression (stages I to IV) according to the revised American Fertility Society classification scheme, suggesting that BDNF and TrKB are correlated with the severity of endometriotic lesions and promote cell proliferation, angiogenesis, and invasion [106]. Moreover, ectopic lesions have been shown to continuously synthesize BDNF, thereby forming a vicious cycle that exacerbates the disease [107].

## 4. Effects of E2 and P4 on NGF and BDNF Expression in Endometriosis

As evidence of the regulation of neurotrophins by E2 and P4 in female reproductive tissue such as the uterus, a study of sexually mature ovariectomized female mice treated with E2 and P4 (subcutaneous administration for three days, followed by a two-day rest period, and then four more days of treatment) found that BDNF mRNA increased with E2 and P4 compared to control animals, while BDNF protein levels were upregulated by E2 [84]. The hypothesis that E2 may modulate the expression and biological effects of neurotrophins is substantiated by the finding that classical EREs are present in the promoter and 5′-flanking regions of the genes for human NGF, p75 (the low-affinity receptor for NGF), TrkA, and BDNF [108,109].

It has been suggested that E2 provides trophic support to nerve fibers in the endometrium and myometrium of women with endometriosis by regulating the production of neurotrophins and other molecules associated with nerve fiber growth [110]. The mechanisms underlying endometriosis-related pain are poorly understood, but estrogen-dependent inflammation may be involved. The immune microenvironment in the peritoneal cavity induces inflammation that may also mediate this pain. Macrophages are among the most abundant immune cells in endometriotic lesions [111]. In peritoneal lesions of women with endometriosis, CD68-positive macrophages have been identified in close association with nerve fibers typical of afferent sensory innervation [13]. In peritoneal endometriotic lesions, all CD68-positive macrophages are REβ-positive, and only 18.3% ± 4.37% are ERα-positive [85]. It was also observed that nerve fibers recruit macrophages in an E2-dependent manner; in turn, E2 increased the neurotrophic properties of macrophages by upregulating BDNF and NT3 expression [85]. Neurogenesis is regulated by macrophages, and this regulation is estrogen-dependent (Figure 2). Macrophages are important sources of NGF. E2 can induce c-Fos expression and shift the composition of the AP-1 complex on the NGF promoter from c-Jun homodimers to c-Fos/c-Jun heterodimers in macrophages. This transformation enhances AP-1 transcriptional activity, leading to increased NGF expression in macrophages [112].

One study found that high levels of E2 in ovarian endometriomas promote the recruitment and activation of mast cells, which release NGF, thereby promoting nerve growth and nerve fiber sensitization. This E2-mediated neuroimmune interaction correlated with dysmenorrhea severity in women with endometriosis compared with women without endometriosis [66].

Dysregulation of estrogen signaling in endometriosis can mediate multiple aspects of the disease, including macrophage recruitment and polarization, neurogenesis, and angiogenesis. The interaction between macrophages and nerve fibers, regulated by estrogens, establishes a vicious circle that exacerbates inflammation in the endometriotic milieu. Persistent inflammatory stimulation of the peripheral nerve fibers results in peripheral sensitization, therefore exacerbating the progression of endometriosis [113].

The therapeutic effects of progestogens used in endometriosis include the inhibition of estrogen synthesis and action, as well as of ER activity; inhibition of the survival and proliferation of endometriotic cells; and suppression of local angiogenesis and neurogenesis [114]. In a mouse model of endometriosis, the role of P4 in inhibiting cell proliferation, inflammation, and neovascularization was investigated. Ovariectomized female mice treated with E2 developed large ectopic endometriotic lesions with abundant blood vessels and extensive adhesions compared with the vehicle-treated group. In contrast, animals treated with E2 in combination with P4 developed smaller, non-vascularized endometriotic lesions than control mice and those treated with E2 alone. In mice treated with E2 followed by P4, there was a significant reduction in Ki67 (a marker of cell proliferation), CD31 (an endothelial marker also known as PECAM-1), and CCN1/Cyr61 (an angiogenic regulator in the pathogenesis of endometriosis and a marker of neovascularization), as well as in the expression of pro-inflammatory cytokines and macrophage infiltration. Interestingly, ERα and PR mRNA expression was downregulated and associated with disease progression, whereas ERβ mRNA gradually increased in the endometriotic lesions of the control animals. Immunohistochemical staining of these lesions also showed a pronounced decrease in ERα and PR protein levels in the control animals [115].

DNG, a synthetic progestin that possesses selective PR agonistic activity and oral progestational potency in the endometrium, is utilized in the treatment of endometriosis [116]. DNG has been reported to downregulate various factors associated with endometriosis pain, including the neuroangiogenic factors such as VEGF and stromal cell-derived factor 1 [117] and NGF [118] in human endometrial epithelial cells. It has been reported that DNG inhibits NGF expression through PR-A and PR-B in human endometrial epithelial cell lines [118] and that NF-κB and AP-1 are involved in this inhibition of NGF expression by DNG. High NGF production in endometriotic foci participates in nerve fiber synthesis and the development of pain symptoms [104,119]. Therefore, the inhibitory effect of DNG treatment on NGF may help improve the pathological condition of patients with endometriosis.

### Clinical Translation

First-line treatments for endometriosis include combined oral contraceptives, progestins, and nonsteroidal anti-inflammatory drugs. For patients whose symptoms are not controlled with first-line agents, second-line suppressive treatments, such as gonadotropin-releasing hormone agonists and antagonists, are offered. Hormonal therapies help reduce pain and may lower the risk of recurrence after surgery; however, their contraceptive effect or the onset of menopause-like symptoms may make them unsuitable for women trying to conceive [120]. This has spurred studies and trials of new medical therapies that aim to provide personalized symptom management while considering women’s desire to become pregnant.

This review provided evidence that coordinated regulation of neurotrophins and hormones promotes the growth and invasion of nerve fibers and blood vessels within endometriotic lesions, which may be associated with the pelvic pain experienced by women with endometriosis. This information is relevant because NGF, BDNF, their Trk receptors, and the signaling pathways they activate (ERK, MAPK, PI3K, and Akt) are potential therapeutic targets, whether administered individually or in combination with hormonal therapies. It is also important to note that new information is emerging regarding the actions of SPRMs and SERMs in endometriosis, which could serve as therapeutic alternatives to existing treatments. A wider variety of therapeutic targets also increases the possibility of applying personalized therapies tailored to the symptoms of patients with endometriosis.

The evidence presented in this review indicates that BDNF levels are significantly higher in patients with endometriosis compared to healthy women [120]. Furthermore, plasma BDNF levels were positively correlated with the severity of pelvic pain in patients with endometriosis [121]. These results suggest that plasma BDNF levels can be used as a diagnostic marker for endometriosis in women with pelvic pain, particularly for detecting ovarian endometriomas (compared to women with other benign ovarian tumors). However, plasma BDNF levels are not useful for indicating the presence of peritoneal endometriosis or deep infiltration [121]. It is important to note that the use of BDNF as a diagnostic marker for endometriosis must consider confounding factors such as the presence of mental disorders or the administration of analgesics, which can alter BDNF levels [120].

Data from a systematic review and meta-analysis indicate that serum NGF levels are higher in patients with endometriosis than in healthy women. These findings suggest that NGF could serve as a biomarker for endometriosis; however, few studies are available, and further research is needed to confirm its utility [96].

An interesting point is that NGF and BDNF are locally synthesized in endometriotic tissue, suggesting that neurotrophins serve as biomarkers for early disease detection. Early diagnosis of endometriosis is a significant challenge due to the confusion surrounding endometriosis symptoms, such as pelvic pain, which is often associated with menstruation discomfort; consequently, women with endometriosis do not receive appropriate treatment [96]. Finding a non-invasive biomarker has been challenging due to disease heterogeneity and comorbidities that can alter its levels [122]. The most consistent finding is that BDNF and NGF levels are significantly higher in the blood of patients with endometriosis. This may contribute to further refinement of measurements and studies of BDNF, NGF, their receptors, the signaling pathways that mediate their effects, and their interactions with ovarian hormones.

## 5. Conclusions

In summary, endometriotic lesions preferentially develop in highly vascularized and innervated environments, underscoring the key roles of angiogenesis and neurogenesis in disease establishment and progression, which are associated with severe pain. These processes are closely linked to the hormonal milieu characteristic of endometriosis, which is characterized by elevated E2 levels and P4 resistance, together disrupting endometrial gene regulation and function.

Emerging evidence highlights a critical interplay between sex steroid signaling and neurotrophins pathways. E2 has been shown to upregulate NGF and BDNF expression in macrophages, mainly via ERβ, thereby promoting neuroangiogenesis associated with lesion growth and pain. Although less studied, P4 also appears to modulate neurotrophins expression, potentially increasing NGF levels in lesion-associated macrophages, whereas progestins such as DNG can suppress NGF expression through PR isoforms. All these findings point to a complex regulatory network linking hormones, neurotrophins, and neurovascular remodeling in endometriosis. A deeper understanding of these interactions could lead to personalized therapeutic strategies for women with endometriosis experiencing severe pain.

## Data Availability

No new data were created or analyzed in this study. Data sharing is not applicable to this article.

## Abbreviations

The following abbreviations are used in this manuscript:

AP-1	Activator protein-1
BMP2	Bone morphogenetic protein 2
BDNF	Brain-derived neurotrophic factor
CGRP	Calcitonin gene-related peptide
cAMP	Cyclic adenosine monophosphate
DNG	Dienogest
E2	Estradiol
ER	Estrogen receptor
ERα	Estrogen receptor α
ERβ	Estrogen receptor β
EREs	Estrogen response elements
ERK1/2	Extracellular signal-regulated kinase 1/2
GPER	G protein-coupled estrogen receptor 1
IL-6	Interleukin-6
IL-8	Interleukin-8
MeSH	Medical subject headings
mPRs	Membrane P4 receptors
NGF	Nerve growth factor
NF-κB	Nuclear factor kappa-B
PI3K	Phosphoinositide-3-kinase
P4	Progesterone
PAQR	Progesterone and adipoQ receptors
PGRMCs	Progesterone receptor membrane components
PR	Progesterone receptor
PR-A	Progesterone receptor-A
PR-B	Progesterone receptor-B
p75NTR	P75 neurotrophin receptor
SERMs	Selective estrogen receptor modulators
SPRMs	Selective progesterone receptor modulators
SMD	Standardized mean difference
Trk	Transmembrane tropomyosin receptor kinase
VEGF	Vascular endothelial growth factor
